# Reconfigurable frequency multipliers based on graphene field-effect transistors

**DOI:** 10.1186/s11671-023-03884-8

**Published:** 2023-10-05

**Authors:** A. Toral-Lopez, E. G. Marin, F. Pasadas, M. D. Ganeriwala, F. G. Ruiz, D. Jiménez, A. Godoy

**Affiliations:** 1 Dpto. Electrónica y Tecnología de Computadores, Facultad de Ciencias, Universidad de Granada, Granada, Spain; 2 Departament d’Enginyeria Electrònica, Escola d’Enginyeria, Universitat Autònoma de Barcelona, Bellaterra, Spain

**Keywords:** Graphene, Split gate, Frequency multiplier, Reconfigurable, Radio frequency, High frequency, Field-effect transistor

## Abstract

Run-time device-level reconfigurability has the potential to boost the performance and functionality of numerous circuits beyond the limits imposed by the integration density. The key ingredient for the implementation of reconfigurable electronics lies in ambipolarity, which is easily accessible in a substantial number of two-dimensional materials, either by contact engineering or architecture device-level design. In this work, we showcase graphene as an optimal solution to implement high-frequency reconfigurable electronics. We propose and analyze a split-gate graphene field-effect transistor, demonstrating its capability to perform as a dynamically tunable frequency multiplier. The study is based on a physically based numerical simulator validated and tested against experiments. The proposed architecture is evaluated in terms of its performance as a tunable frequency multiplier, able to switch between doubler, tripler or quadrupler operation modes. Different material and device parameters are analyzed, and their impact is assessed in terms of the reconfigurable graphene frequency multiplier performance.

**Supplementary information:**

The online version contains supplementary material available at (10.1186/s11671-023-03884-8).

## Introduction

Leveraging ultra-high carrier mobility and saturation velocity, graphene has been, along the last two decades, extensively investigated as an outstanding alternative for high-frequency (HF) electronics, showing almost on par performance with cutting-edge III–V technologies [1]. Its striking transport properties are complemented by its inherent carrier ambipolarity, exhibited by a V-shaped transfer characteristics around the Dirac voltage. The combination of these features in the same material opens the path to the exploration and conception of novel HF designs able to greatly simplify and overcome conventional circuits.

One of the most suited and, at the same time, unexplored HF modules that could harness graphene properties are frequency multipliers. A frequency multiplier is a device/circuit that, under ideal operation, generates a single-frequency harmonic at a desired multiple of the frequency of an input signal. The physical implementation of this function is not straightforward and relies upon the nonlinear characteristic of a device which distorts the input signal, generating a spectrum of harmonics, which are later band-pass filtered. State-of-the-art frequency multipliers are mostly based on Schottky diodes with relatively high efficiencies but lacking amplification and with limited frequency selectivity [2]. Although the continuous technological optimization, both at the device architecture and at the III-V material quality, has pushed the operation of III-V diode-based frequency multipliers to the frontiers of the THz realm [3], novel concepts, exploiting distinct physics and designs, are susceptible to become game changers for the HF field in the hundreds of GHz [4] and THz ranges [5].

Graphene field-effect transistors (GFETs) exhibit the potential ingredients to break through the limitations of Schottky diodes and have indeed proved to be a good test bed to implement frequency doublers [6] reaching frequencies in the GHz range [7–9]. The series connection of two GFETs with conveniently tuned Dirac points has also been demonstrated as a powerful design to obtain higher harmonics such as triplers [10, 11] and quadruplers [12, 13] with notable conversion efficiencies. The main idea is to combine several V-shaped ambipolar transfer characteristics to attain a W-shaped response, which properly adjusted produces the required nonlinearity to generate the desired output harmonic. The physical implementation of this W-shaped characteristic can be achieved under different device architectures: (i) at the circuit-design level, connecting several independent GFETs, or (ii) more interestingly, at the device-design level, using independent gates to control the same channel and selectively adjust the device operation mode [14]. This latter case involves the additional appealing concept of extending the device functionality, i.e., reconfiguring the device beyond the implementation of a single function, aiming to get a higher functional density with a constant integration density [15].

In this regard, the combination of a back- and top-gate is the most employed configuration [12]. However, it has demonstrated a limited frequency performance due to the large parasitic capacitance originated by the global back-gate [13]. To overcome this serious drawback, we propose and analyze a split-gate GFET architecture able to perform as a reconfigurable frequency multiplier generating $\times 2$, $\times 3$ or $\times 4$ harmonics of the input signal, with electrically controllable frequency reconfigurability under operating conditions. The analysis is carried out making use of a physically based simulator that includes a detailed description of the channel material through its density of states (DoS) integrated in the time-dependent transport equation [16–18], considering both, DC and transient analysis.

The rest of the paper is organized as follows: first, discusses the ideal response that can be expected from frequency multipliers, and its resemblance with the W-shaped transfer characteristic obtained in GFETs by tuning the Dirac voltage, is discussed; next, the numerical simulator is described and validated through comparison with experimental results; then, the main outcomes of this study are presented, followed by the conclusions extracted from them.

## Response of ideal frequency multipliers

**Fig. 1:**
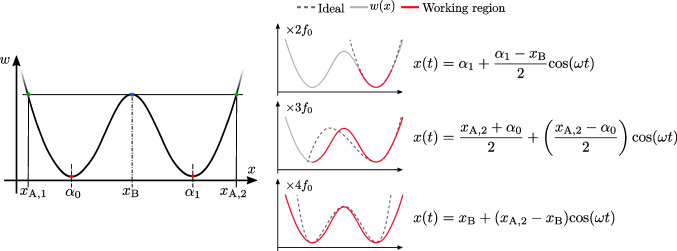
Comparison of the W-shaped profile of Eq. (1) (gray lines) and the ideal profiles (dashed lines) for the ×2, ﻿﻿×3 and ×4 frequency multiplication. The corresponding working regions of *w*(*x*) to obtain each frequency multiplication factors are indicated in red, along with the corresponding input signals to operate in each region *x(t)*. These expressions for the input signals depend on the five main parameters of *w*(*x*) indicated at the bottom part of the figure.

The theoretical expression for ideal frequency multiplication, i.e., single-harmonic generation from a sinusoidal input, is a polynomial function where the eventual multiplication factor is determined by the order of the polynomial (see Supporting Information for details). For the case of frequency doubling, tripling and quadrupling, one can find a good resemblance in the shape of these ideal functions with different regions of a W-shaped function (see Fig. 1).

This W shape can, indeed, be achieved as the addition of two shifted quadratic responses, which is the ideal relationship between gate bias ($V_\textrm{gs}$) and drain current ($I_\textrm{ds}$) in GFETs, and a constant factor, which would correspond to a bias-independent conductive region or element connecting the two hypothetical GFETs. The resulting W-shaped response, and, to a good level of approximation, the series association of two GFETs (or any other architecture emulating this association), can thus be analytically described by the following expression:

1 $$\begin{aligned} w(x) =\left[ \frac{1}{\beta _{0}(x-\alpha _{0})^{2} + \gamma _{0}} + \frac{1}{\beta _{1}(x-\alpha _{1})^{2} + \gamma _{1}} + \frac{1}{\gamma _{2}}\right] ^{-1} \end{aligned}$$

where $\alpha _{0}$ and $\alpha _{1}$ correspond to the position of the first and second minima (i.e., the Dirac voltages of the two GFETs ); $\beta _{0}$ and $\beta _{1}$ set the curvature of each parabola (i.e., the transconductance of the GFETs); and $\gamma _{0}$ and $\gamma _{1}$ are a vertical shift of each parabola (i.e., they set the minimum conductivity at the Dirac voltages), while $\gamma _{2}$ is a constant factor (i.e., the conductance of the element connecting both GFETs). Note that, from a circuit theory perspective, Eq. (1) just puts down a series association of three conductive regions, that would in principle correspond to two channels of graphene connected by a constant conductivity region.

Figure 1 shows the comparison between the W-shaped function (solid light gray) in Eq. (1) and the ideal theoretical response for $\times 2$, $\times 3$ and $\times 4$ frequency multiplication (dashed lines). Depending on the desired response, a different working region of *w*(*x*) (solid red) must be selected by modifying the bias and amplitude of the input signal *x*(*t*). The actual amplitude and bias values of the input signals for frequency doubling, tripling and quadrupling are defined in Fig. 1.

The frequency spectra (see Additional file 1) of the different curves demonstrate the appropriateness of *w*(*x*) to generate the required frequency multiplication, with a slightly smaller amplitude than the ideal response and with some small-amplitude spurious at integer multiples of the input frequency.

## Numerical simulation scheme and validation

In order to move the theoretical discussion to a practical design, and more interestingly, to devise new concepts and architectures able to exploit graphene for frequency multiplication, it is necessary to determine to what extent the actual response of the GFETs is able to emulate the ideal W shape in Eq. (1). To this purpose, we exploit a simulation suite that encompasses the self-consistent solution of the Poisson equation along with the time-dependent continuity equation under a semiclassical dissipative umbrella, a reasonable approach considering typical device sizes. The range of applicability of the presented model is limited to the appearance of propagating electromagnetic waves in the device and the necessary consideration of the complete form of time-dependent coupled Maxwell equations. The electrostatic potential and the charge density profile in a 2D cross-section of the structure are related through the Poisson equation:

2 $$\begin{aligned} \nabla \cdot \left( \varepsilon \,\,\overrightarrow{\nabla } V\right) = -\rho \end{aligned}$$

where $\varepsilon$, *V* and $\rho$ are the permittivity, potential and charge distribution, respectively. All of them are defined in the XY plane, and the structure is considered invariant along the *z*-axis. The term $\rho$ includes the fixed charge associated with impurities as well as mobile carriers, i.e., electrons and holes. The time-dependent continuity equation is written as:

3 $$\begin{aligned} \nabla \cdot \left( \textbf{J} + \frac{\partial \textbf{D}}{\partial t}\right) = 0 \end{aligned}$$

where $\textbf{D}$ is the electric displacement field and $\textbf{J}$ the current density along the channel, expressed in terms of the Fermi-level gradient as [19]: $J = (\mu _{n}n + \mu _{p}p)\nabla E_\textrm{F}$, where $\mu _{n}$ $(\mu _{p})$ is the electron (hole) mobility dependent on the longitudinal electric field [20], and *n* (*p*) the electron (hole) carrier densities. The Fermi level is solved using the continuity equation and combined with the conduction and valence band profiles to self-consistently evaluate the surface carrier densities as:

4 $$\begin{aligned} \begin{aligned} n(x)&= \int \mathrm{{DoS}_\textrm{n}}(E)f_\textrm{FD}(E_\textrm{D}(x)-E_\textrm{F}(x))dE\\p(x)&= \int \mathrm{{DoS}_\textrm{p}}(E)\left[ 1-f_\textrm{FD}(E_\textrm{D}(x)-E_\textrm{F}(x))\right] dE \end{aligned} \end{aligned}$$

where $\textrm{DoS}_\textrm{n}$ ($\textrm{DoS}_\textrm{p}$) is the conduction (valence) band DoS, $f_\textrm{FD}$ the Fermi–Dirac function, and $E_\textrm{D}$ stands for the Dirac energy determined by the electrostatic potential obtained solving the Poisson equation. The DoS profiles can be provided by either external *ab-initio* calculations or theoretical expressions such as the graphene linear DoS profile: $\mathrm{{DoS}} = q^2{2|E|}/{\pi (\hbar v_\textrm{F})^2}$, where $v_\textrm{F} = 10^8$ cm/s is the Fermi velocity in graphene. The values for $E < 0$ correspond to $\mathrm{{DoS}_\textrm{p}}$, while $E > 0$ to $\mathrm{{DoS}_\textrm{n}}$. For stationary simulations, we solve the same self-consistent set of equations, neglecting the term $\frac{\partial D}{\partial t}$ in Eq. (3). A more detailed description of the iterative scheme and the integration of the equations can be found in the Additional file 1.

**Fig. 2:**
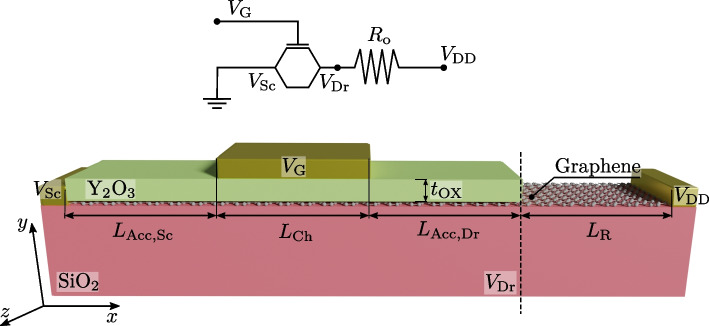
Device used to validate the simulator from the experimental data in Ref. [6]. An underlap graphene region has been included to model a series resistor $R_\textrm{o}$ with the aim of reproducing the same circuit used in its characterization (top panel)

To validate the proposed numerical approach, we make use of the data provided in Ref. [6], where a graphene FET with a $L_\textrm{Ch}=500$ nm-long gate and $L_\textrm{Acc, Sc} = L_\textrm{Acc, Dr}=500$ nm-long source and drain access regions was fabricated (see Fig. 2), with the graphene layer deposited on a SiO$_2$ substrate and a Y$_2$O$_3$ layer as gate insulator, with $t_\textrm{ox}=5$ nm. Notably, the experimental circuit is rather clear and simple, enabling the direct comparison with the measurements with minimal post-processing of the numerical simulation outputs, avoiding complex de-embedding procedures that could complicate the study of the single device response and thus impact in the soundness of the validation. The experimental topology included a series resistor ($R_{\rm o}$), as shown in the upper section of Fig. 2, emulated in the numerical simulations with a $L_\textrm{R} =500$ nm-long additional region of constant conductivity. The resistance value of this region is given by $R_\textrm{o}|_\mathrm{Sim.} = \int _{L_\textrm{R}}{\frac{dx}{q\mu _\textrm{R}(n+p)}}$ where $\mu _\textrm{R} = 140$ cm$^2$/Vs was set to achieve a $R_\textrm{o}|_\mathrm{Sim.} \approx 9$k$\Omega$, mimicking the experimental resistance value.

**Fig. 3:**
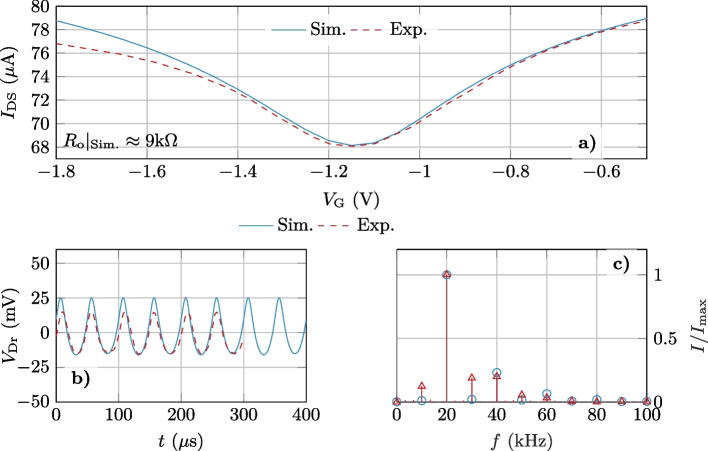
**a** Comparison of the experimental $I_\textrm{DS} - V_\textrm{G}$ characteristic [6] (dashed) with the numerical one (solid) for the device in Fig. 2. **b** Experimental (red) and computed (blue) output ($V_\textrm{Dr}$) obtained for a 10kHz input signal applied on the gate and **c** their spectra normalized by the maximum achieved at $f=20$ kHz

We first focused on the experimental transfer characteristic, represented as a dashed line in Fig. 3a. We tuned the carrier mobilities in the channel ($\mu _\textrm{n} = \mu _\textrm{p}=465$ cm$^2$/Vs), the residual carrier density due to puddles ($N_\textrm{puddles}=11.8\cdot 10^{11}$ cm$^{-2}$) and the carrier mobility in the resistive region. The dielectric constant of the gate insulator and the difference between the metal-gate and graphene work function $q(\phi _\textrm{m} - \chi _\textrm{gr})$ were set to $12.6\,\varepsilon _0$ and $-0.375$ eV, respectively. The result of the fitting is depicted as solid line in Fig. 3a, proving the capability of the numerical simulator to reproduce the electrical response of the device.

Once the DC behavior was assessed, we dealt with time-dependent simulations to reproduce the experiments carried out with a 10 kHz input signal and 400 mV of amplitude, employing the same tuned parameters. The experimentally obtained output waveform and its spectrum are depicted in Fig. 3b (red) jointly with the simulated signal (blue). A very good agreement is achieved between both signals, with the amplitude of the $\times$ 2 harmonic clearly outperforming the rest of components in the output spectra shown in Fig. 3c, stressing the capability of the approach here developed to reproduce the experimental response.

## Results and discussion

Next, we assessed the capabilities of GFETs to emulate the ideal W-shaped transfer characteristic and therefore to perform as frequency multipliers. To this aim, we propose a novel architecture based on a split-gate configuration where the quadratic conductance of the GFETs is exploited in combination with a controlled Dirac voltage shift (DVS) [16].

**Fig. 4:**
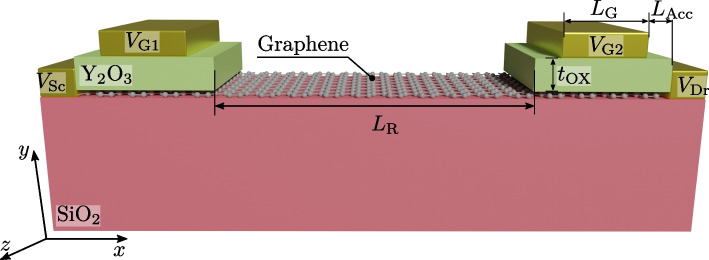
Structure used to implement the frequency multiplier defined by two channels serially connected by an intermediate uncontrolled region. The geometry of the device is defined by $L_\textrm{R}=530$ nm, $L_\textrm{G}=115$ nm, $L_\textrm{Acc}=10$ nm and $t_\textrm{OX}=20$ nm.

The structure is depicted in Fig. 4, and it comprises a single graphene layer deposited on top of a SiO$_2$ substrate. The graphene is partially covered by a split gate with two contacts ($V_\textrm{G1}$ and $V_\textrm{G2}$), both with length $L_\textrm{G}=115$ nm, each of them located at one edge of the graphene layer. The gate metals underlap a Y$_2$O$_3$ insulating layer of thickness $t_\textrm{OX}=20$ nm and length 135 nm (resulting in two *L*_Acc_= 10 nm long underlapped access regions at both sides of each gate). The split-gate configuration gives rise to two channels controlled by two independent gates and connected in series through an extended graphene layer of $L_\textrm{R}=530$ nm. The conductivity of this region is not modulated by any gate (i.e., there is not back-gate in the proposed architecture), and it acts as a resistor causing a voltage drop between the drain of the first channel and the source of the second. This potential drop results in a distinct DVS at each graphene channel, determined by the corresponding drain and source biases [21], giving rise to the W shape.

**Fig. 5:**
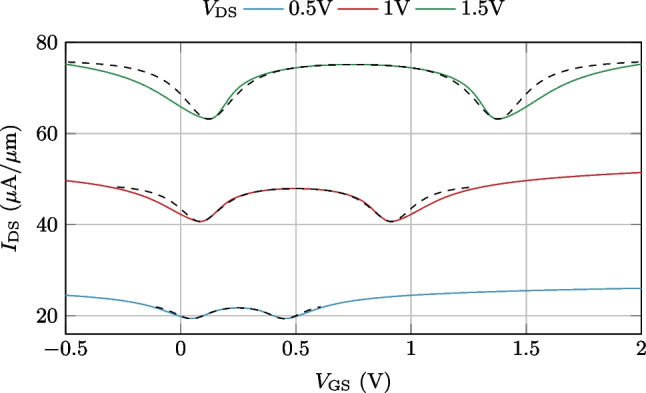
$I_\textrm{DS}-V_\textrm{GS}$ characteristics of the split gate device for three different $V_\textrm{DS}$ values and V_GS_ = V_G1_ = V_G2_. The black dashed lines indicate the fitting with the function $w(V_\textrm{GS})$, with $\alpha _0 = V_\textrm{Dirac,1}$ and $\alpha _{1} = V_\textrm{Dirac,2}$

Figure 5 depicts the transfer characteristic of the split-gate architecture for $V_\textrm{G1}=V_\textrm{G2}=V_\textrm{GS}$, and three values of $V_\textrm{DS}$: 0.5V (blue), 1V (red) and 1.5V (green). The $I_\textrm{DS}-V_\textrm{GS}$ characteristics depict two minima, indicating that the split-gate GFET results in distinct DVS in the two channels. In order to assess its resemblance with the theoretical W shape, the curves were fitted with the function $I_\textrm{DS}=w(V_\textrm{GS})$ in Eq. (1), with $\alpha _{0}=V_\textrm{Dirac,1}$ and $\alpha _{1}=V_\textrm{Dirac,2}$, and tuning $\beta _{0}$, $\beta _{1}$, $\gamma _{0}$, $\gamma _{1}$ and $\gamma _{2}$, for each $V_\textrm{DS}$. The resulting values are summarized in Table 1. The symmetry of both channels is evidenced by the similarity of $\beta _{0}$ and $\beta _{1}$ as well as $\gamma _{0}$ and $\gamma _{1}$. As can be observed, $V_\textrm{DS}$ directly affects the Dirac voltage values ($V_\textrm{Dirac,1}$ and $V_\textrm{Dirac,2}$) and their difference. The later can, indeed, be adjusted with $V_\textrm{DS}$, which sets an upper limit to $V_\textrm{Dirac, 2}-V_\textrm{Dirac, 1}$. Unfortunately, $V_\textrm{DS}$ also impacts in the shape of the W (as revealed by the variations in $\beta _{i}$ and $\gamma _{i}$) generating a notable change in the device output current. Figure 5 proves the limitations for a proper W-shaped generation by solely varying the DVS of both channels through $V_\textrm{DS}$.

An alternative method to control the position of both Dirac points (and to generate the W shape) with a lower impact in the output current is to independently bias each gate. A gate bias difference ($\Delta V_\textrm{G} = V_\textrm{G2} - V_\textrm{G1}$) directly translates into different DVS in both channels, as if they would have distinct metal work functions [21]. With this methodology, one can expect a controlled change in the position of both Dirac points with a lower impact in the output current. Indeed, $\Delta V_\textrm{G}$ can be interpreted as an electrically *operando* reconfiguration knob, converting the split-gate GFET architecture in a reconfigurable device able to implement different frequency multiplication factors as we will discuss in the following.

**Table 1:** Values of the parameters used to fit the transfer characteristics shown in Fig. 5 using the function *w*(*x*)

$V_\textrm{DS}$	$\beta _{0}$	$\gamma _{0}$	$V_\textrm{Dirac,1}$	$\beta _{1}$	$\gamma _{1}$	$V_\textrm{Dirac,2}$	$\gamma _{2}$
0.5V	8.62	0.081	0.046V	8.79	0.081	0.453V	15.64
1V	8.18	0.099	0.085V	8.25	0.1	0.914V	20.57
1.5V	5.44	0.095	0.122V	5.47	0.095	1.377V	19.78

**Fig. 6:**
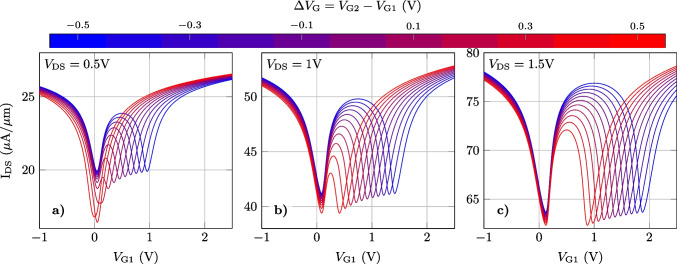
$I_\textrm{DS}-V_\textrm{G1}$ characteristics of the device for three different $V_\textrm{DS}$ values: **a** 0.5 V, **b** 1 V, **c** 1.5 V, as a function of the control bias $\Delta V_\textrm{G}$. For each curve, $V_\textrm{G2}$ corresponds to $V_\textrm{G1}$ plus a constant value $\Delta V_\textrm{G}$ according to the upper color bar

First, in order to assess the capability of $\Delta V_\textrm{G}$ to generate a W-shaped response in the split-gate GFET architecture, we have calculated the $I_\textrm{DS}-V_\textrm{G1}$ characteristics for different values of $\Delta V_\textrm{G}$, spanning in a range from −0.5 V to +0.5 V, and for three different $V_\textrm{DS}$ values: 0.5 V, 1 V and 1.5 V, respectively. The results are depicted in Fig. 6 indicating that for a fixed $V_\textrm{DS}$ value, $\Delta V_\textrm{G}$ provides a large tunable adjustment of the DVS without incurring in a substantial variation of the current. In more detail, $\Delta V_\textrm{G}$ is able to increase the distance between both minima well above the applied $V_\textrm{DS}$, which is the upper limit achieved in Fig. 5. The current values at both minima differ moderately for the lowest $V_\textrm{DS}=0.5$ V, but they are almost equal for $V_\textrm{DS}=1.5$ V. Interestingly, the Dirac voltages become closer when $\Delta V_\textrm{G}$ approaches to the applied $V_\textrm{DS}$ bias, and notably, it is even possible to merge both minima with an appropriate value of $\Delta V_\textrm{G} = V_\textrm{DS}$, as illustrated in the case $V_\textrm{DS} = 0.5$ V. This feature becomes attractive for switching from a W shape to V shape, namely the *operando* change of the frequency multiplication between $\times$4$f_\textrm{in}$ and $\times$2$f_\textrm{in}$.

**Fig. 7:**
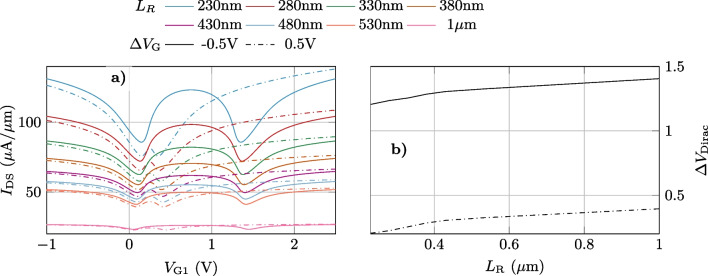
**a** $I_\textrm{DS}-V_\textrm{G1}$ for different lengths of the intermediate region $L_\textrm{R}$ for two different values of $\Delta V_{G}$, $-$0.5 V (solid) and 0.5 V (dot-dashed), and a constant $V_\textrm{DS}=1$V. **b** $\Delta V_\textrm{Dirac}=V_\textrm{Dirac,1}-V_\textrm{Dirac,2}$ *vs* $L_\textrm{R}$

The graphene region connecting both channels also plays a key role in the controlled separation between both Dirac points and the W shape generation. In order to gain insights into this point, we extend the analysis of the transfer function to assess the impact of $L_\textrm{R}$ on the device characteristics. Figure 7 shows $I_\textrm{DS}-V_\textrm{G1}$ for two $\Delta V_\textrm{G}$ values, $-$0.5 V (solid) and 0.5 V (dashed), and $V_\textrm{DS}=1$ V, for different values of $L_\textrm{R}$ in a range from 230 nm to 1 $\mu$m. The output current is clearly impacted by $L_\textrm{R}$, as the resistance of this intermediate region scales up with the length. In other words, a lower resistance in this region (lower $L_\textrm{R}$) improves the overall conductivity of the structure, enabling a higher output current. A slight variation in the separation of the minima ($\Delta V_\textrm{Dirac}=V_\textrm{Dirac,1}-V_\textrm{Dirac,2}$) as a function of $L_\textrm{R}$ is shown in Fig. 7b. This effect might be related to the impact of the diffusion length at the edge of the channels [22]. For $L_\textrm{R} < 0.4\,\mu$m, the potential and charge distribution of the channels has a significant influence on the intermediate region. This in turn involves a higher modulation of the $V_\textrm{DS}$ bias of the channels, providing a large variation of their Dirac points.

*Time-dependent simulations*

**Fig. 8:**
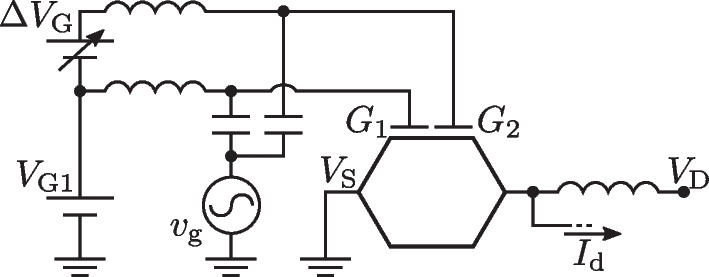
Setup employed to analyze the frequency performance of the split-gate GFET. Two independent DC voltage sources, $V_\textrm{G1}$ and $\Delta V_\textrm{G}$, are used to set the bias point and the $I-V$ characteristic, and a small-signal source $v_\textrm{g}$ feeds both gates

Once the DC characteristics of the device have been analyzed, we perform time-dependent simulations to assess its AC response when a sinusoidal signal is used as input. This study is carried out making use of the setup depicted in Fig. 8, with two independent DC gate biases used to select the device working region and an AC input signal $v_\textrm{g}$ feeding both gates simultaneously.

**Fig. 9:**
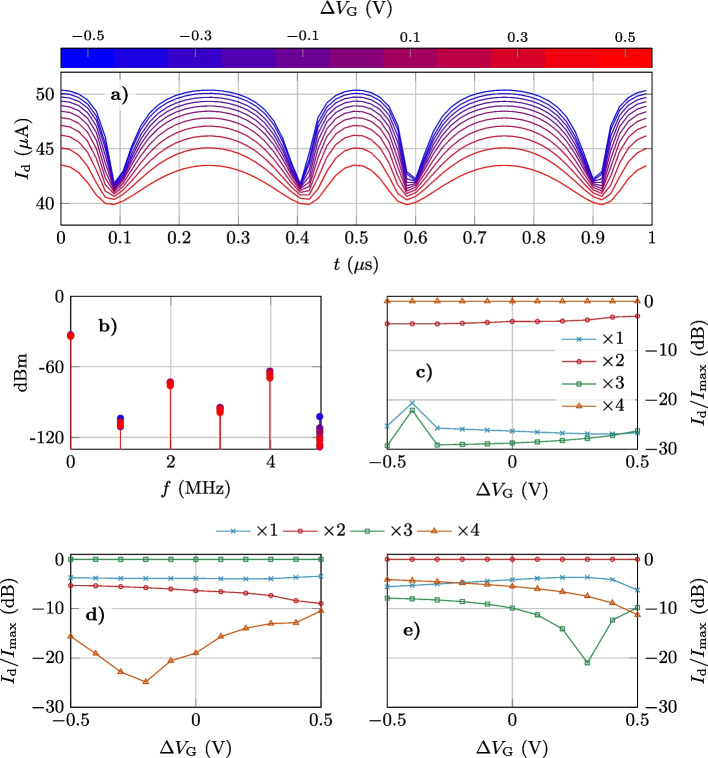
**a** Transient output current for a constant $V_\textrm{DS}=$1V, for ten different values of $\Delta V_\textrm{G}$ ranging from −0.5V to +0.5V and $V_\textrm{G1}$ at the mid-point between the Dirac points. **b** Output power spectrum for a 1MHz sinusoidal input ($v_\textrm{g}$) assuming a 50$\Omega$ load. **c** Normalized amplitude of the output harmonics as a function of $\Delta V_\textrm{G}$ when the device operates as a frequency tripler **d** and as a frequency doubler **e**

We assume $V_\textrm{DS}=1$ V, while $\Delta V_\textrm{G}$ is swept from −0.5 V to +0.5 V, and $V_\textrm{G1}$ is tuned to be centered in the mid-point between $V_\textrm{Dirac,1}$ and $V_\textrm{Dirac,2}$, e.g., for $\Delta V_\textrm{G}=0.5$ V, $V_\textrm{G1}\simeq 0.25$ V as shown in Fig. 6b. For that polarization, an AC input signal is applied to the gates with frequency $f_\textrm{in}=1$ MHz and the required amplitude to operate as a frequency quadrupler as indicated in Fig. 1. Figure 9a shows the drain current as a function of time for different $\Delta V_\textrm{G}$ biases, and Fig. 9b and c their corresponding frequency spectrum with the different normalized harmonic amplitudes. As expected, the device output shows a main harmonic at $\times$4$f_\textrm{in}$. It must also be stressed the noticeable contribution of the $\times$2$f_\textrm{in}$ harmonic, which lays $<5$ dB below the main tone ($\times$4$f_\textrm{in}$). The device response agrees with the theoretical prediction (Additional file 1), and it is a result of the capability of the split-gate architecture to reproduce the ideal W behavior. Regarding the impact of $\Delta V_\textrm{G}$ on the spectral purity, the contribution of the $\times$2$f_\textrm{in}$ harmonic is slightly reduced as $\Delta V_\textrm{G}$ becomes more negative, while the odd harmonics (with a notable smaller amplitude) increase. Therefore, the $\Delta V_\textrm{G}$ makes possible to modify the weight of the different harmonics. This feature is shown in Fig. 9d and e, where the split-gate is configured to operate as a tripler (Fig. 9d) or as a doubler (Fig. 9d). In each case, the third and second harmonic, respectively, becomes dominant, with some modulation of the relative amplitudes of the rest of harmonics with $\Delta V_\textrm{G}$. The operation of the split-gate GFET as a frequency doubler and tripler is discussed in more detail in Additional file 1, proving that the device can be reconfigured to operate as an efficient doubler or tripler through the control of $V_\textrm{G1}$ and $\Delta V_\textrm{G}$.

*Operation frequency*

**Fig. 10:**
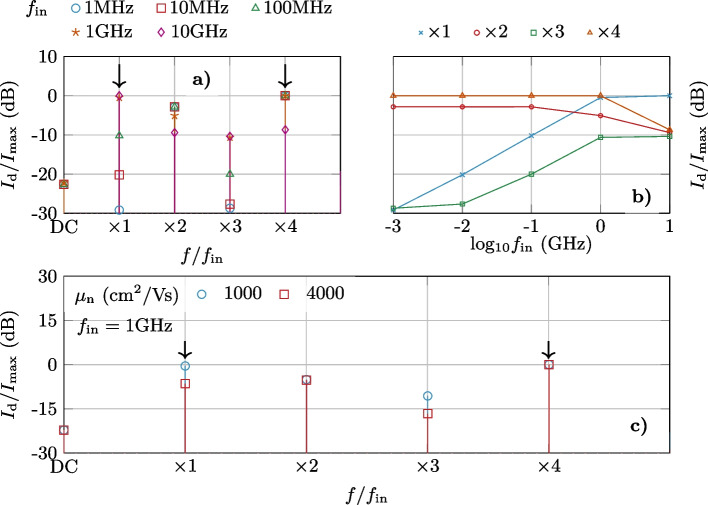
**a** and **b** Output spectrum for different $f_\textrm{in}$ and $\Delta V_\textrm{G} = 0.5$ V. **c** Variation of the first four harmonics when the electron mobility increases for $f_\textrm{in}=1$ GHz

Once the AC operation of the split-gate GFET as a reconfigurable frequency multiplier has been assessed, we proceed with a prospective study of the maximum operation frequency achievable by the device. We focus on the quadrupler, as it is the scenario where the limitation would be more critical. We assume $V_\textrm{DS} = 1$ V and $\Delta V_\textrm{G} = 0.5$ V, although similar conclusions could be drawn for different values of both biases. $V_\textrm{G1}$ is set at the mid-point between both Dirac points, and the frequency of $v_\textrm{g}$ is swept in a range from 1 MHz to 10 GHz, while its amplitude is kept as in Fig. 9. The obtained output spectrum is depicted in Fig. 10a, b for the first four harmonics normalized to the maximum for different frequencies.

While the $\times 4$ harmonic is still dominant for sub-GHz frequencies, the contribution of the input frequency $f_\textrm{in}$ in the device output increases exponentially, reaching the $\times 4$ harmonic at $f_\textrm{in} = 1$ GHz and becoming dominant for $f_\textrm{in} = 10$ GHz. This is due to the capacitive leaking through the gate insulator of the AC signal and gives insights about the maximum input frequency for a proper operation of the frequency multiplier. It is worth to note that this limit is uniquely determined by the gate oxide stack and might be improved reducing the capacitive coupling. Moreover, the relative contribution of the displacement and diffusion current is also impacted by the carrier mobility. Figure 10c shows the frequency spectrum at $f_\textrm{in} = 1$ GHz for two different values of symmetrical electron/hole mobilities: 1000 cm$^2$/Vs and 4000 cm$^2$/Vs ($\mu _\textrm{n} = \mu _\textrm{p}$). The results demonstrate that the contribution of the $\times$4$f_\textrm{in}$component in the output is raised with an increasing mobility, resulting in 6.5 dB gain with respect to the $\times 1$ harmonic that was similar in amplitude for the lower mobility of 1000 cm$^2$/Vs. Notably, an improved mobility also reduces the $\times 3$ harmonic amplitude.

## Conclusions

A split-gate graphene FET architecture is presented as a reconfigurable frequency multiplier, leveraging the addition of two ambipolar quadratic $I-V$ characteristics. The split-gate GFET operation is thoroughly analyzed and its performance as reconfigurable frequency doubler, tripler and quadrupler is assessed in terms of the output spectra. It is demonstrated that the selection of the appropriate bias point on both independent gates results on the dynamic reconfiguration of the device operation. Moreover, the impact of the diffusive and displacement currents is discussed, proving the beneficial effect that an improved carrier mobility produces on the maximum operating frequency achievable by the device. The frequencies of these devices are still lower than the current off-the-shelf technologies. However, graphene is still a teenage material in a non-mature technological stage, with large potential for improvement and eventually overcome well-established semiconductors. This work adds to those demonstrating that beyond direct comparison of RF performance, graphene technology enables single-device implementation of complex nonlinear responses. The proposed architecture opens the path to the exploration of novel designs exploiting unmatched graphene ambipolarity and its inherent high-mobility in reconfigurable high-frequency electronics.

### Supplementary information

**Additional file 1.** Supplementary figures.

## Data Availability

The output data of the simulations carried out on this work are available from the corresponding author under reasonable request.

## Declarations

### Code availability

The source code is available upon reasonable request from the corresponding author.
