# Supplementary material for: Reconfigurable frequency multipliers based on graphene field-effect transistors
Source: Discov Nano. 2023 Oct 5;18(1):123. doi: 10.1186/s11671-023-03884-8 (PMC10555978; doi:10.1186/s11671-023-03884-8)
Supplement: Supplementary file 1 — Additional file 1. Supplementary figures. [file 11671_2023_3884_MOESM1_ESM.pdf]

Supporting Information: Reconfigurable frequency multipliers based on graphene field-effect transistors

A. Toral-Lopez,[1, a)] E.G. Marin,[1] F. Pasadas,[1] M. D. Ganeriwala,[1] F.G. Ruiz,[1] D. Jiménez,[2] and A. Godoy[1, b)]

[1)] *Dpto. Electrónica y Tecnología de Computadores, Facultad de Ciencias, Universidad de Granada (Spain)*

[2)] *Departament d'Enginyeria Electrònica, Escola d'Enginyeria, Universitat Autònoma de Barcelona, Bellaterra (Spain)*

(Dated: 27 July 2023)

1. Ideal response of frequency multipliers

The output of an ideal frequency multiplier would correspond to a single-harmonic tone at a multiple of the input signal frequency. The theoretical function to generate this output is a polynomial whose order is given by the multiplication factor. Here we briefly discussed the $\times 2 f_{\text{in}}$, $\times 3 f_{\text{in}}$ and $\times 4 f_{\text{in}}$ ideal theoretical functions.

For the first case, to double the input signal frequency, a quadratic transfer characteristic $f_{\times 2}(x) = x^2$ could be employed to generate a two-fold factor in the output frequency without additional components except for a constant DC value:

$$f_{\times 2}(A\cos(\omega t)) = \frac{A^2}{2}(1 + \cos(2\omega t)) \tag{1}$$

To triple the input frequency, a function $f(x) = x^3$ is required that would also generate an additional linear component:

$$\begin{aligned} f_{\times 3}(A\cos(\omega t)) &= A^3\cos^3(\omega t) \\ &= \frac{A^3}{4}(3\cos(\omega t) + \cos(3\omega t)) \\ &= \frac{3}{4}A^3\cos(\omega t) + \frac{A^3}{4}\cos(3\omega t) \end{aligned} \tag{2}$$

Therefore, it is necessary to combine this x^3 with the appropriate linear term, i.e. $f_{\times 3}(x) = x^3 - \frac{3}{4}A^2 x$, to obtain the ideal behavior.

Regarding the response of the frequency quadrupler, the x^4 function needs to be combined with the appropriate quadratic element ($f_{\times 4}(x) = x^4 - A^2x^2$) to eliminate undesired harmonics in the output (except for a DC component):

$$\begin{aligned} f_{\times 4}(A\cos(\omega t)) &= A^4\cos^4(\omega t) - A^2(A^2\cos^2(\omega t)) \\ &= \frac{A^4}{4}(\cos(2\omega t) + 1)(\cos(2\omega t) + 1) - A^4\cos^2(\omega t) \\ &= \frac{A^4}{4}(\cos^2(2\omega t) + 2\cos(2\omega t) + 1) - A^4\cos^2(\omega t) \\ &= \frac{A^4}{4}\left(\frac{\cos(4\omega t)}{2} + 2\cos(2\omega t) + \frac{3}{2}\right) - A^4\cos^2(\omega t) \\ &= \frac{A^4\cos(4\omega t)}{8} + \frac{A^4\cos(2\omega t)}{2} + \frac{3A^4}{8} - A^4\cos^2(\omega t) \\ &= \frac{A^4\cos(4\omega t)}{8} + \frac{3A^4}{8} + \frac{A^4\cos(2\omega t)}{2} - \\ &- \frac{A^4\cos(2\omega t) + A^4}{2} = \frac{A^4\cos(4\omega t)}{8} - \frac{A^4}{8} \end{aligned} \tag{3}$$

The functions $f_{\times 2}(x)$, $f_{\times 3}(x)$ and $f_{\times 4}(x)$, are plotted in the left column of Figure S1 (blue line) along with $w(x)$ in Eq. (1) of the main text (red line) and their spectrum in each case (right column) for an input $x = A\cos(2\pi f_{in} t)$.

Multiplication factor	Input
$\times 2$	$\frac{\alpha_1 - x_{\text{B}}}{2}\cos(\omega t) + \alpha_1$
$\times 3$	$\left(\frac{x_{\text{A},2} - \alpha_0}{2}\right)\cos(\omega t) + \frac{x_{\text{A},2} + \alpha_0}{2}$
$\times 4$	$(x_{\text{A},2} - x_{\text{B}})\cos(\omega t) + x_{\text{B}}$

TABLE SI. Input signals considered to obtain the spectrums in Figure S1. The amplitude and bias of the signals are defined according to the five points highlighted in Figure 1 of the main manuscript.

[a)] Electronic mail: atoral@ugr.es
[b)] Electronic mail: agodoy@ugr.es

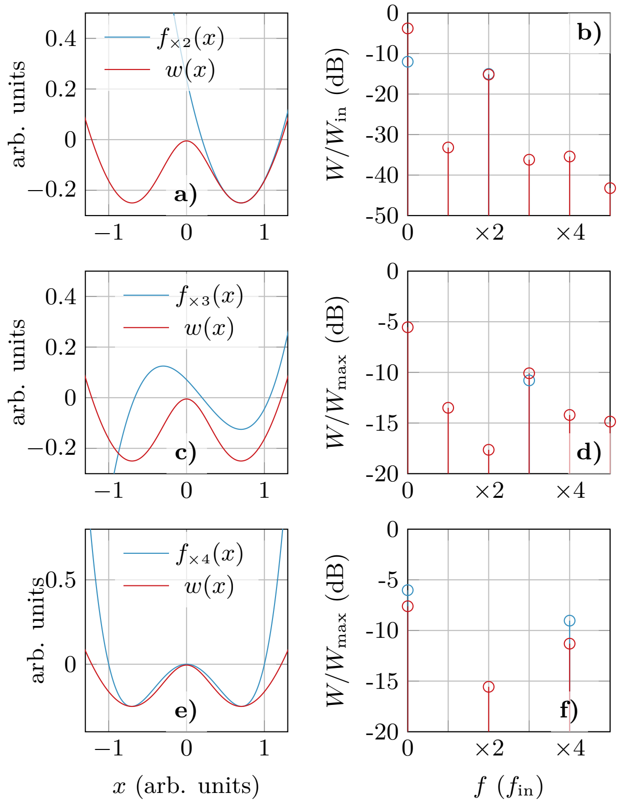

FIG. S1. (Left column) Comparison between $w(x)$ (red line) and the ideal responses $f_{\times 2}(x)$, $f_{\times 3}(x)$ and $f_{\times 4}(x)$ (blue line). (Right column) Spectrum of $w(x)$ and $f_{\times i}(x)$ (×2 b), ×3 d), ×4 f)) when a $\cos(\omega t)$ signal is considered as the input. In the case of $w(x)$, the input signal is adjusted according to Table SI to select the proper operation region.

2. $f_{\times 4}(x)$ as arbitrary frequency multiplier

The W-shaped profile of the $f_{\times 4}(x)$ function can be harnessed to operate not only as quadrupler but also as a tripler and a doubler. Due to its similarity with $w(x)$ in Eq. (1) of the main manuscript, the operation regions depicted in Figure 1 of the main manuscript can be applied to this case along with Table SI. Figure S2 depicts in red the output spectrum obtained when $f_{\times 4}(x)$ is used to generate a two-fold S2a and three-fold S2b factor in the output frequency. These results are compared with the spectrum produced by the ideal response (green) in each scenario, demonstrating that a quadrupler can also be employed as a tripler or doubler providing a noticeable performance.

3. Operation of the split-gate GFET as doubler and tripler

As previously indicated, $w(x)$ can be used to implement $\times 2 f_{\rm in}$ and $\times 3 f_{\rm in}$ frequency multipliers using the appropriate input signal amplitude and biases. In order to test this conclusion on the device under study, we again make use of the expressions in Table SI to set the input signals for each case.

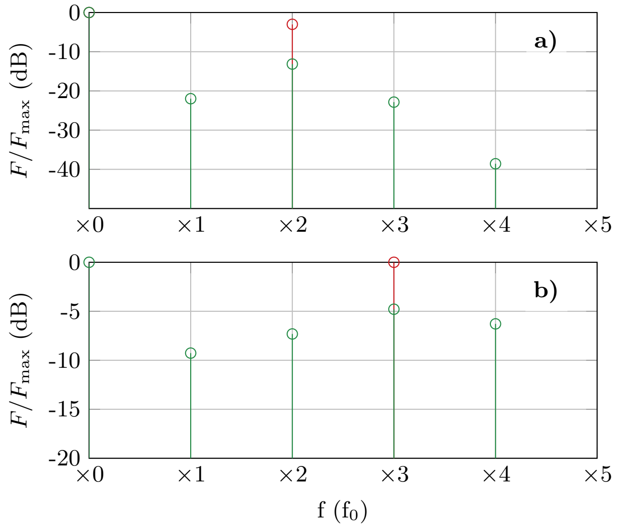

FIG. S2. Response of the ×4 frequency multiplier (green) when operating as a ×2 a) and ×3 b) frequency multiplier compared with the ideal response (red) for each case.

To simulate the operation as a tripler the input signal is biased to $V_{\rm G1} = (x_{\rm A,2} + V_{\rm Dirac,1})/2$ with an amplitude $A = x_{\rm A,2} - V_{\rm Dirac,1}/2$. The results are depicted in Figures S3a and S3c, showing that, among all the components of the output signal, the $\times 3 f_{\rm in}$ harmonic has the largest contribution, with $>$ 3dB above the first harmonic. This indicates that the device is operating properly as a $\times 3 f_{\rm in}$ frequency multiplier. The output spectrum shows that the contribution of the odd components (×1 and ×3) barely changes when $\Delta V_{\rm G}$ is modified, while the contribution of the even components ($\times 2 f_{\rm in}$ and ×4) are prone to be altered by $\Delta V_{\rm G}$, mainly the $\times 4 f_{\rm in}$ component.

With regard to the $\times 2 f_{\rm in}$ frequency multiplier, the device is reconfigured to work around one of the Dirac points of the curve. In this case we select $V_{\rm Dirac,1}$, so $V_{\rm G} = V_{\rm Dirac,1}$ and $A = (V_{\rm C} - V_{\rm Dirac,1})/2$. The output signals obtained for this case and their spectrums are depicted in Figures S3b and S3d.

Now, the main component of the output is the desired $\times 2 f_{\rm in}$ component, with $>$ 3dB above the first harmonic. In contrast to the case of the tripler, we observe a significant change in the contribution of the rest of harmonics as $\Delta V_{\rm G}$ is modified. Nevertheless, the amplitude of the input signal will determine the suitable $\Delta V_{\rm G}$, similar to the case of the $\times 4 f_{\rm in}$frequency multiplier.

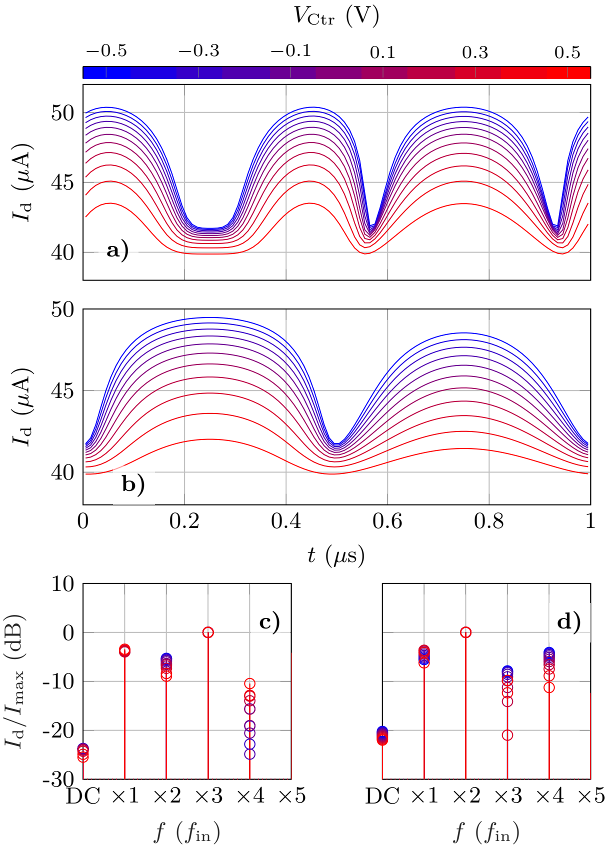

FIG. S3. Transient output current signal and its spectrum when the device operates as a (a, c) $\times 3 f_{\mathrm{in}}$ and a (b, d) $\times 2 f_{\mathrm{in}}$ frequency multiplier. Color code is refereed to ΔV_{G} value indicated on the top.

4. Numerical simulation scheme

Figure S4 shows the iterative scheme followed to solve the equations that describe the electrostatics (Poisson equation) and charge transport (Continuity and Drift-Diffusion equations) in the device. First, the 2D electrostatic potential across the structure (V) is obtained. Next, it is provided to the transport equations which are used to obtain the profile of the Fermi level, which is subsequently employed to estimate the electron and hole profiles (n and p). In this step V is used to obtain the displacement current ($\partial D/\partial t$) and the Dirac point along the channel (E_{D}). The transport equations are only evaluated in the longitudinal direction as we assume that the vertical component of the current is almost null due to the high confinement of carriers in these materials. This equation provides a profile for the Fermi level that is later combined with E_{D} and the density of states to obtain the longitudinal carrier density profiles. Finally, the 1D carrier density profiles are extended in the vertical direction using a sinusoid-shaped profile to obtain the 2D profile of the net charge density. Then, this carrier concentration is used to obtain a new solution of V. This process is repeated until convergence on the electrostatic potential is achieved.

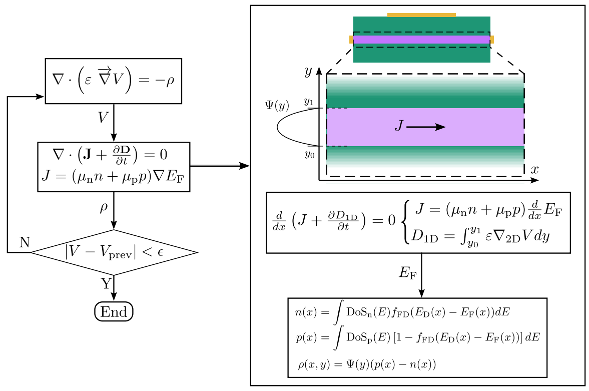

FIG. S4. Scheme of the iterative algorithm employed in the numerical simulations. The main core of the calculations is based on the self-consistent solution of the Poisson and continuity equations. This latter one includes both the electric and displacement currents. As depicted in the right-hand side of the figure, no transport is assumed in the vertical direction due to the high confinement of 2D materials. Therefore, the continuity equation is defined only along the device length. This equation is used to obtain the profile of the Fermi level, which is subsequently employed to estimate the electron and hole profiles.

References
